# *Scandinavium lactucae* sp. nov. Isolated from Healthy Lettuce in South Korea

**DOI:** 10.1007/s00284-024-03811-9

**Published:** 2024-08-07

**Authors:** Jiwon Park, Sieun Park, Kwang-Kyo Oh, Charles M. A. P. Franz, Gyu-Sung Cho

**Affiliations:** 1 Department of Microbiology and Biotechnology, Max Rubner-Institut, Federal Research Institute of Nutrition and Food, Hermann-Weigmann-Straße 1, 24103 Kiel, Germany; 2 Microbial Safety Division, National Institute of Agricultural Sciences, Rural Development Administration, Wanju, 55365 Republic of Korea

## Abstract

Four Gram-negative, facultative anaerobic, oxidase-negative and catalase-positive strains were isolated from lettuce sample collected from test beds at the National Institute of Agricultural Sciences in Wanju, South Korea. The whole genome sequences of the strains ranged from 4,624,629 to 4,849,846 bp in size, with DNA G + C contents of 54.32 to 54.56 mol%. Phylogenetic analyses based on 16S rRNA gene and four housekeeping (*atp*D, *gyr*B, *inf*B, and *rpo*B) gene sequences showed that the four strains clustered closely together with *Scandinavium* type strains within the *Enterobacteriaceae* family. Moreover, the average nucleotide identity and digital DNA-DNA hybridization value of the proposed type strain (V105_6^T^) with the closely related *Scandinavium* type strains were in the range of 85.71–86.16% and 30.2–31.2%, respectively, which were all below the species delineation threshold values. The major cellular fatty acid of V105_6^T^ was C_16:0_. Growth was observed at 7, 10 and 35 °C, and in the presence of 7% NaCl concentration. Based on phenotypic and genotypic results, strain V105_6^T^ represents a novel species of the genus *Scandinavium*, for which the name *Scandinavium lactucae* sp. nov. is proposed. The type strain is V105_6^T^ (= LMG 33389^T^ = DSM 117134^T^).

**Supplementary Information:**

The online version contains supplementary material available at 10.1007/s00284-024-03811-9.

## Introduction

The *Enterobacteriaceae* family represents a diverse group of Gram-negative bacteria, which are facultative anaerobic, oxidase-negative and catalase-positive, and have been isolated from various environments including water, soil and the intestinal tracts of humans and animals, where they can lead to a variety of infections [1]. Numerous commensal and pathogenic species within this family possess antibiotic resistance genes and harbor mobile genetic elements that can facilitate the transfer of antibiotic resistance determinants [2]. For this reason, antibiotic-resistant *Enterobacteriaceae* are emerging as global public health threats and pose major challenges in the treatment of infections [3].

In a previous study, the novel genus *Scandinavium* was added to the family *Enterobacteriaceae* [2]*.* The novel species within this genus, named *Scandinavium* (*S.*) *goeteborgense,* was isolated from a wound infection of an adult patient in Sweden and it carries a quinolone resistance gene variant (*qnrB*96) which exhibited high resistance to the antibiotic ciprofloxacin. This single strain was identified by sequencing of the complete 16S rRNA gene and whole genome sequencing analyses, including average nucleotide identity (ANI) and in silico DNA-DNA similarity analysis [2]. In 2022, three additional species, namely *Scandinavium hiltneri*, *Scandinavium manionii* and *Scandinavium tedordense* were described within the *Scandinavium* genus [4]. The type strains of *S. hiltneri* H11S7^T^ and *S. manionii* H17S15^T^ were isolated from *Quercus robur* rhizosphere soil in Hatchlands, Guildford, United Kingdom, while the type strain of *S. tedordense* TWS1a^T^ was obtained from a bleeding lesion on a *Tilia x europaea* tree in Tidworth, Wiltshire, United Kingdom [4].

The global consumption of fresh produce has significantly risen, reflecting an increasing trend in dietary preferences worldwide. However, consuming it raw exposes individuals to potentially harmful bacteria, including antibiotic-resistant. With the increasing demand for fresh produce and the growing preference for raw consumption, understanding the microbial composition and antibiotic resistance profiles of lettuce is crucial for assessing public health risks and ensuring food safety [5].

In the present study, four strains obtained from lettuce exhibited high 16S rRNA gene sequence similarity to species of the *Scandinavium* genus. Additionally, multi locus sequence analysis (MLSA), overall genome related indices (ANI and dDDH), and whole genome-based phylogeny of these strains together with closely related type strains in this genus indicated that the isolates clearly differed from other *Scandinavium* species type strains and represented a novel species, for which the name *S. lactucae* is proposed.

## Materials and Methods

### Strain Isolation and Propagation

All strains were isolated from healthy lettuce (*Lactucae sativa*) sample in the same batch collected from test beds at the Korean National Institute of Agricultural Sciences (NAS) between 29 June and 28 July 2021 on violet red bile dextrose agar (VRBD; Merck, Darmstadt, Germany) plates [6]. All strains were routinely cultured in Luria–Bertani (LB; Roth, Karlsruhe, Germany) broth at 35 °C for 18 h. For long-term storage, the isolates were stored in the same medium at −70 °C with 25% (v/v) glycerol.

### Phenotypic Characterization and Cell Morphology

Colony morphology was determined on LB (Roth) agar after incubation at 35 °C for 18 h. Strains were incubated further at 7, 10, 35, and 41 °C for up to 120 h to determine the optimal temperature range for growth. To test salt tolerance, stains were inoculated in LB (Roth) broth with NaCl concentrations ranging from 1 to 10% and were incubated at 35 °C for up to 120 h. The pH tolerance of the strains was tested by adjusting the pH of tryptone soy broth (TSB, Oxoid) from 4 to 12 using 1 N hydrochloric acid (Roth) and 1 N sodium hydroxide (Roth), followed by incubation at 35 °C for 120 h. The miniaturized identification system ID 32 E (BioMerieux, Nürtingen, Germany) was used for phenotypic identification based on biochemical reactions such as enzyme activities and sugar fermentations. For this, all isolates were first sub-cultured on a LB (Roth) agar plate at 35 °C for 18 h. A single colony was picked, suspended in 0.85% NaCl with turbidity adjusted to 0.5 McFarland. Each test strip was inoculated with the suspension and incubated per the manufacturer’s instructions.

### Phylogenetic and Genomic Analyses

To extract total genomic DNA of the isolates, the peqGOLD bacterial DNA extraction kit (VWR, Darmstadt, Germany) was used according to the manufacturer's instructions. DNA concentration and quality were measured with a Qubit 3 fluorometer (Invitrogen, Darmstadt, Germany) and a NanoDrop spectrophotometer (Peqlab, Erlangen, Germany).

Random amplified polymorphic DNA-PCR (RAPD-PCR) was performed using primer M13, and repetitive sequence-based PCR (rep-PCR) used the (GTG) 5 primer [6, 7]. BioNumeric software (v. 8.0; Applied Maths, Belgium) was applied for the clustering analysis of RAPD-PCR and rep-PCR using Pearson correlation coefficient and Dice coefficient, respectively.

The Trimmomatic pipeline (v. 0.32; parameters: Phred 33, slidingwindow; 4:15, leading; 3, and minlen; 45) trimmed Nextseq adapter sequences from raw data [8], and *de-novo* genome assembly was conducted using SPAdes (v. 3.15.5) with the ‘isolate’ parameter with quality checked by QUAST (v. 5.0.2) [9, 10]. Contigs shorter than 500 bp and PhiX contaminated contigs were removed using the bbmap and bbduk pipelines (BBTools (v. 38.90)—DOE Joint Genome Institute). Contigs were annotated by the BV-BRC (Bacterial and Viral Bioinformatics Resource Center) server (v. 3.30.19) [11], Prokka (v. 1.14.0) [12] and the NCBI PGAP (Prokaryotic Genome Annotation Pipeline) version 4.13 [13]. CheckM analysis (v. 1.2.2) was performed using the *Enterobacteriaceae* CheckM marker set on the NCBI PCAP gene set [14], and PlasmidFinder (database: 2023–01-18) identified known plasmid replication types [15] with default parameters.

The almost complete 16S rRNA gene sequences of each isolate were obtained using Sanger sequencing (Microsynth, Göttingen, Germany) and analyzed with EzBioCloud [16] for identification of the isolates. A phylogenetic tree of 16S rRNA gene sequences was constructed with MEGA (v. 10) [17] using maximum-likelihood method and Kimura two-parameter model [18] along with 1000 bootstrap analysis. The four housekeeping genes, ATP synthase beta subunit (*atp*D), DNA gyrase subunit B (*gyr*B), initiation translation factor 2 (*inf*B), and RNA polymerase beta subunit (*rpo*B) were extracted from whole genome sequence data and multilocus sequence analysis (MLSA) was conducted with the type strains of the previously described species of the genus *Scandinavium* and other type strains of the family *Enterobacteriaceae* using the MEGA (v. 10) program [17]. The maximum-likelihood method and Kimura two-parameter model were applied, with bootstrap values based on 1000 replications. Additionally, a phylogenetic tree based on whole genome sequences was reconstructed with the types strains of previously described species of the genus *Scandinavium*, as well as selected *Enterobacteriaceae* type strains, using the BV-BRC server [11]. For this, 100 conserved coding sequences were extracted and concatenated. A homologous group filtering and sequence alignment were performed using MAFFT pipeline which was implanted to BV-BRC, and a phylogenetic tree was generated using the RAxML pipeline with 100 bootstrap values. In order to identify all isolates precisely, average nucleotide identity (ANI) and *in-silico* DNA-DNA hybridization (dDDH) values were calculated using OrthoANI (v. 0.93.1) [19] supported by BLAST tool [20] and the Genome-to-Genome Distance Calculator (v. 3.0) with formula 2, respectively [21]. A heatmap was generated using R (v. 4.3.2.) [22] to assess clonal relationship among isolates.

### Antibiotic Resistance Test

All isolates were sub-cultured on a LB (Roth) agar plate at 35 °C for 18 h. A single colony was then suspended in Mueller–Hinton broth (Roth) and incubated at 35 °C for 18 h. For the disc diffusion test, strains were spread on a Mueller–Hinton (Roth) agar plates and incubated with antibiotic discs at 35 °C for 18 h. In the test, ampicillin (AMP) 10 µg, cefoxitin (FOX) 30 µg, cefotaxime (CTX) 30 µg, chloramphenicol (C) 30 µg, ciprofloxacin (CIP) 5 µg, gentamicin (CN 10) 10 µg, erythromycin (E) 15 µg, meropenem (MEM) 10 µg, sulfonamide (S) 10 µg, tetracycline (TE) 30 µg, and tobramycin (TOB) 10 µg containing discs were tested. Th inhibition zone diameters were measured, and susceptibility was determined to be susceptible, intermediate or resistant according to the Clinical & Laboratory Standards Institute (CLSI, 2020) guidelines [23].

### Cellular Fatty Acid Analysis

Analysis of the cellular fatty acid profile of the novel species candidate V105_6^T^ was carried out commercially by the DSMZ (Deutsche Sammlung von Mikroorganismen und Zellkulturen) (Braunschweig, Germany). Briefly, cells were grown on LB agar plate at 35 °C for 18 h. The cellular fatty acids were converted into fatty acid methyl esters (FAMEs) by saponification and methylation, and extracted according to instruction’s manual of the Microbial Identification System (MIDI Inc.; v. 6.1) [24]. The FAMEs were separated by a GC–MS run on an Agilent GCMS-7000D system, detected by a flame ionization detector, and identified using Sherlock Microbial Identification System (TSBA; v. 6.1 library) [25].

### Virulence Gene Identification

ABRicate (v. 1.0.1) was utilized for screening virulence genes using the virulence factor database (VFDB; 2024-05-10) [26] under default parameters. The Type VI Secretion System (T6SS) was further investigated using the SecReT6 database, which includes bioinformatically predicted T6SS gene cluster nucleotide data (v. 3.0; 2021-11-15) [27]. The protein sequence similarity of the pentapeptide repeat QnrB family protein from the isolates was compared with QnrB1 and QnrB96 using BLASTp with default parameters [28].

## Results and Discussion

### Colony Morphology and Phenotypic Characteristics

*Scandinavium* sp. strain V105_6^T^ formed cream colonies of 1–2 mm size when incubated at 35 °C for 18 h on LB (Roth) agar. The colony shape, margin, and elevation of all strains were circular, entire, and flat, respectively. All strains were Gram-negative and exhibited growth at temperatures of 7, 10, and 35 °C, while no growth was observed at 41 °C. They showed tolerance to concentrations ranging from 1–7% NaCl, with particularly weak growth in the presence of 7% NaCl. In addition, the growth of all isolates was observed in TSB (Roth) at a pH range of 4–11, while strain V105_16 exhibited no growth at pH 4.

### Phylogenetic and Genomic Analyses

The whole genome sequencing results are summarized in Table 1. The genomes of strains V105_1, V105_6^T^, V105_12 and V105_16 comprised 32, 40, 42, and 45 contigs with a total length of between 4,645,037 bp (V105_12) and 4,854,890 bp (V105_1). The coding sequences ranged from 4,286 bp (V105_16) to 4,517 bp (V105_1), with a mol G + C content ranging from 54.32% (V105_1 and V105_6^T^) to 54.56% (V105_12 and V105_16). The GenBank and SRA accession numbers for the strains are shown in Table 1.

**Table 1:** Summary of whole genome sequencing results of *Scandinavium* isolates from lettuce

	V105_6^T^	V105_1	V105_12	V105_16
No. of contigs	40	32	42	45
N_50_	270,399	676,478	271,596	239,271
G + C content (%)	54.32	54.32	54.56	54.56
Total length (bp)	4,822,990	4,849,846	4,624,629	4,624,648
Genome coverage	× 100	× 59	× 104	× 112
No. of CDS	4513	4517	4288	4286
No. of tRNAs	49	80	55	53
No. of rRNAs	6	15	6	6
Pseudogenes	46	45	56	54
CheckM completeness	100	100	100	100
CheckM contamination	1	1	1	1
The highest 16S rRNA sequence similarity (%)	*S. manionii* (99.39)	*S. manionii* (99.45)	*S. manionii* (99.39)	*S. manionii* (99.39)
Phenotypic antibiotic resistance	AMP, FOX (CIP)	AMP (CIP, FOX)^a^	AMP, FOX	AMP, FOX (CTX)
Plasmid sequence(s)	n.d	n.d.^b^	ColRNAI	n.d
SRA accession no	SRX24351625	SRX24351624	SRX24351626	SRX24351627
GenBank accession no	JAWXRD000000000	JAWXRE000000000	JAWXRC000000000	JAWXRB000000000

^a^Intermediate resistance

^b^n.d., not detect

The partial 16S rRNA gene sequences of these isolates exhibited pairwise sequence similarities of 99.39% to 99.45% similarity to other *Scandinavium* type strains, as determined by the EzBioCloud pipeline (Table 1). Phylogenetic analysis based on 16S rRNA gene sequences grouped these isolates closely with *Scandinavium* type strains, and were clearly separated from the other type strains within the *Enterobacteriaceae* family, except *Pluralibacter gergoviae*, the closest related species to the *Scandinavium* genus (Suppl. Figure 1). Additionally, RAPD-PCR and rep-PCR fingerprinting of these isolates revealed highly similar fingerprint profiles between V105_1 and V105_6^T^, and V105_12 and V105_16 (Suppl. Figure 2).

**Fig. 1:**
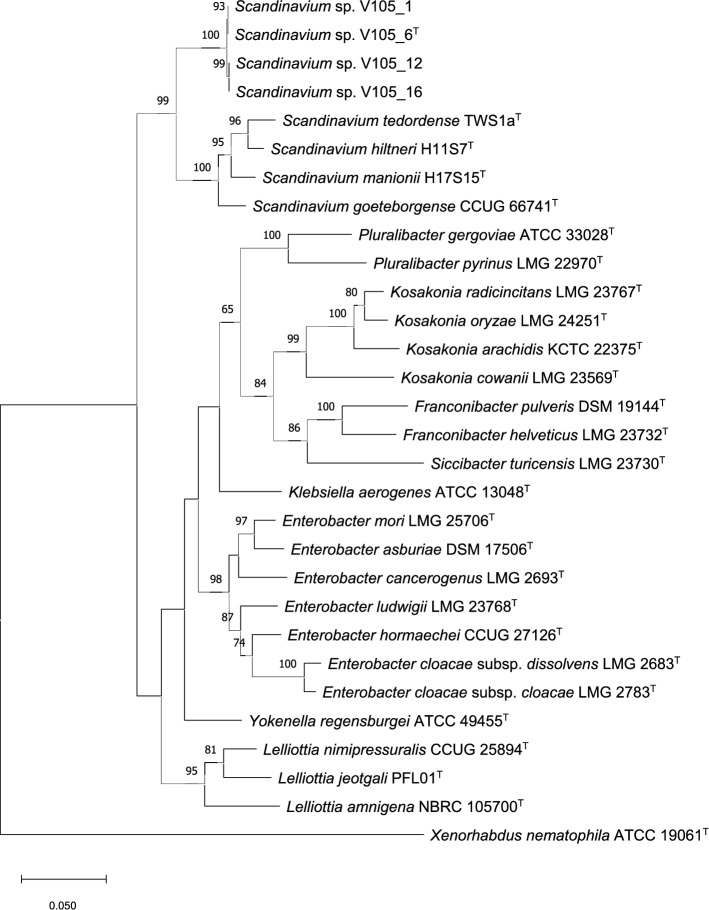
Phylogenetic tree of *Scandinavium* isolates with closely related type strains of the family *Enterobacteriaceae* based on concatenated housekeeping gene sequences of *atpD*, *gyrB*, *infB*, and *rpoB*. The outgroup included *Xenorhabdus nematophila* ATCC 19061^T^. Bootstrap values (> 50%) for 1000 replicates are indicated at branch points, while the scale bar represents the number of substitutions per site

Based on MLSA phylogenetic analysis, including type strains of *Scandinavium* species like *S. goeteborgense* CCUG 66741 T*, S. hiltneri* H11S7T, *S. manionii* H17S15T, and *S. tedordense* TWS1aT, all lettuce isolates were closely related to each other. They showed closer affinity to *Scandinavium* species type strains than to those of *Pluralibacter* species. Importantly, they formed a distinct group separate from all *Scandinavium* species type strains, indicating a novel species (Fig. 1). ANI and dDDH values of all four isolates were below the species delineation thresholds (< 70% for dDDH, < 96% for ANI) when compared to other *Scandinavium* spp. type strains (Table 2) [29]. Additional genome sequences related to our isolates were searched in NCBI database using 6 housekeeping genes (16S rRNA, *atp*D, *dna*J, *inf*B, *gyr*B, and *rpo*B) by BLASTn, and the top-100 related genome sequences were extracted. However, dDDH analysis results indicated that there were no further related whole genome sequences among these strains. The highest ANI value (86.29%) was between strain V105_12 and *S. goeteborgense* CCUG 66741^T^, and the lowest (85.47%) was between strain V105_16 and *S. tedordense* TWS1a^T^ (Table 2). Notably, all four isolates showed the same highest dDDH value of 31.1% similarity compared to *S. goeteborgense* CCUG 66741^T^, and the lowest value of 29.8% was for V105_12 and V105_16 compared to *S. tedordense* TWS1a^T^ (Table 2).

**Table 2:** Average Nucleotide Identity (ANI) and in silico DDH (dDDH) values were determined between *Scandinavium* novel species candidates and closely related type strains

ANI (%) dDDH (%)	1	2	3	4	5	6	7	8
1. V105_6^T^	–	99.99	99.27	99.25	86.19	86.05	85.97	85.71
2. V105_1	100.0	–	99.23	99.22	86.16	86.05	85.99	85.71
3. V105_12	94.4	94.4	–	100.0	86.29	85.93	86.00	85.51
4. V105_16	94.4	94.4	100.0	–	86.26	85.86	85.92	85.47
5. *S. goeteborgense* CCUG 66741^T^	31.2	31.2	31.1	31.1	–	93.10	93.21	92.38
6. *S. hiltneri* H11S7^T^	30.7	30.8	30.7	30.7	52.1	–	95.38	94.61
7. *S. manionii* H17S15^T^	30.6	30.7	30.6	30.6	52.3	63.5	–	94.05
8. *S. tedordense* TWS1a^T^	30.1	30.2	29.8	29.8	48.7	59.0	56.0	–

**Fig. 2:**
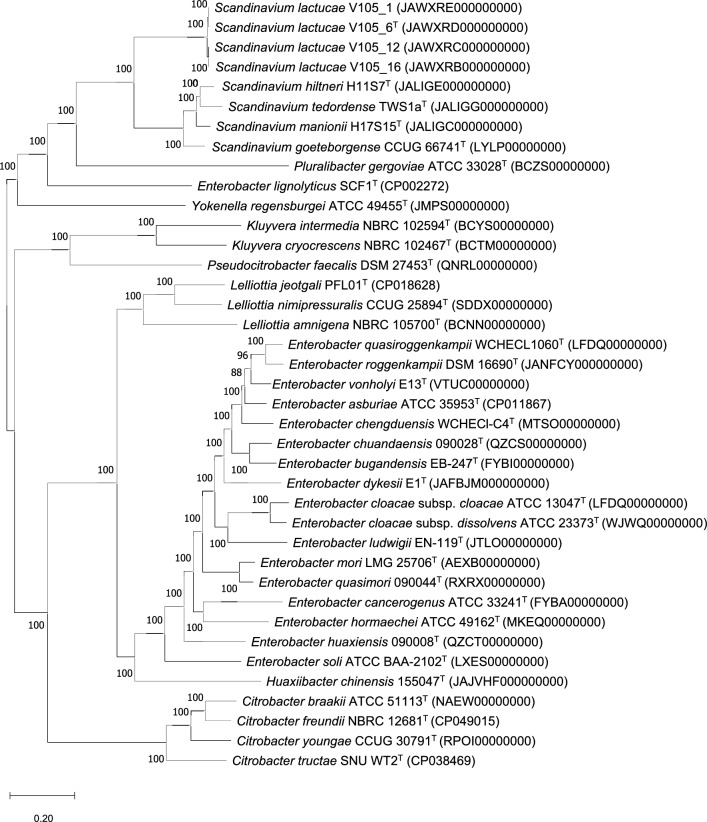
Phylogenetic tree based on whole genome sequences of strains V105_1, V105_6^T^, V105_12, and V105_16 with closely related *Scandinavium* type strains including some *Enterobacteriaceae* type strains. The tree was constructed by extracting and concatenating 100 conserved coding sequences using BV-BRC server [11]. The MAFFT pipeline which was implanted to BV-BRC was used for a homologous group filtering and sequence alignment. Numbers at nodes indicate the percentage of 1000 bootstrap replications. Bar, 0.20 substitutions per nucleotide position

The accession numbers for the whole genome sequence data are indicated in Table 1 and Fig. 2. ANI and dDDH were calculated using the OrthoANI [19] and the Genome-to-Genome Distance calculator (formula 2) [21], respectively

Furthermore, a heatmap based on accessory genes (PANAROO v. 1.4.2) [30] indicated that V105_1 and V105_6^T^, and V105_12 and V105_16 are clonal isolates (Suppl. Figure 3), supporting the RAPD-PCR and rep-PCR fingerprinting results. The whole genome phylogenetic tree clustered the four strains closely with *Scandinavium* type strains, distinct with type strains of other genera within the family *Enterobacteriaceae* such as *Pluralibacter* and *Enterobacter* (Fig. 2). Altogether, these findings indicated that strain V105_6^T^ could be regarded as a novel species in the *Scandinavium* genus.

### Phenotypic Characterization

All four strains tested positive for ornithine decarboxylase, lysine decarboxylase, β-glucosidase, malonate, β-galactosidase (ONPG), and α-glucosidase. Acid production was observed from galacturonate, D-mannitol, D-maltose, D-glucose, L-arabinose, D-trehalose, L-rhamnose, D-cellobiose, and acidification of phenol red (Table 3). In contrast, Other *Scandinavium* type strains were negative for ornithine decarboxylase, malonate and α-glucosidase. *S. hiltneri* H11S7^T^ was the only type strain that tested negative for lysine decarboxylase, while *S. manionii* H17S15^T^ was the only type strain that tested positive for α-galactosidase (Table 3).

**Table 3:** Phenotypic profiles of all known members of the genus *Scandinavium*. Other type strains data were adapted from previous study [6]

Characteristic	V105_6^T^	V105_1	V105_12	V105_16	*S. goeteborgense* CCUG 66741^T^	*S. hiltneri* H11S7^T^	*S. manionii* H17S15^T^	*S. tedordense* TWS1a^T^
Ornithine decarboxylase	+	+	+	+	-	-	-	-
Arginine dihydrolase	-	-	-	-	-	-	-	-
Lysine decarboxylase	+	+	+	+	+	-	+	+
Urease	-	-	-	-	-	-	-	-
L-arabitol	-	-	-	-	-	-	-	-
Galacturonate	+	+	+	+	+	+	+	n.d
Potassium 5-ketogluconate	-	-	-	-	-	-	-	-
Lipase	-	-	-	-	-	-	-	n.d
Phenol red	+	+	+	+	+	+	+	n.d
Β-Glucosidase	+	+	+	+	+	+	+	n.d
D-Mannitol	+	+	+	+	+	+	+	+
D-Maltose	+	+	+	+	+	+	+	+
D-Adonitol	-	-	-	-	-	+	+	+
Palatinose	-	-	-	-	-	-	-	n.d
Β-Glucuronidase	±	+	+	±	-	-	-	n.d
Malonate	+	+	+	+	-	-	-	n.d
Indole production	-	-	-	-	-	-	-	-
N-Acetyl-β-glucosaminidase	-	-	-	-	-	-	-	n.d
Β-Galactosidase (ONPG)	+	+	+	+	+	+	+	+
D-Glucose	+	+	+	+	+	+	+	+
Saccharose	-	-	-	-	-	-	-	-
L-Arabinose	+	+	+	+	+	+	+	+
D-Arabitol	-	-	-	-	-	+	+	+
Α-Glucosidase	+	+	+	+	-	-	-	n.d
Α-Galactosidase	-	-	-	-	-	-	+	n.d
D-Trehalose	+	+	+	+	+	+	+	+
L-Rhamnose	+	+	+	+	+	+	+	+
Inositol	-	-	-	-	-	-	-	-
D-Cellobiose	+	+	+	+	+	+	+	+
D-Sorbitol	±	±	±	±	-	-	+	-
Α-Maltosidase	-	±	±	-	-	-	-	n.d
L-Aspartic acid Arylamidase	-	-	-	-	-	-	-	n.d

+ ; positive, -; negative, ± ; ambiguous, n.d.; not determined

### Antibiotic Resistance

The antibiotic resistance profile of the isolates was determined by the disc diffusion method following CLSI guidelines [23]. The results showed that all four strains were resistant to ampicillin, while all strains except V105_1 were resistant also to cefoxitin. Strain V105_1 exhibited an intermediate response to this antibiotic. Moreover, an intermediate resistance occurred for both strains V105_6^T^ and V105_12, while strain V105_1 showed an intermediate response to cefotaxime (Suppl. Table 1). According to the BLASTp results, the QnrB family protein sequences of all four isolates were identical. When compared to the novel quinolone resistance pentapeptide repeat protein QnrB96, they showed 95.79% sequence identity with 9 aa (amino acid) substitutions, while QnrB1 exhibited 86.92% sequence identity with 28 aa substitutions (Suppl. Figure 4). This indicates that the four isolates share significant similarity with QnrB96, which was identified in *S. goeteborgense* CCUG 66741^T^.

### Fatty Acid Methyl Ester Analysis

The principal fatty acids identified included C_12:0_, C_14:0_, C_16:0_, C_18:1_ *ω7c*, summed feature 2 (C_14:0_ 3-OH and/or iso-C_16:1_) and summed feature 3 (C_16:1_ *ω7c* and/or C_16:1_ *ω6c*). All cellular fatty acid profiles for *Scandinavium* species type strains and potential novel species candidates were indicated in Table 4. The fatty acid compositions of strain V105_6^T^ and V105_16 exhibited remarkable similarity with strains of previously examined species of *Scandinavium.*

**Table 4:** The average percentage peaks and standard deviations of the major fatty acid methyl esters (FAME) were recorded for strain *S*. *lactucae* V105_6^T^, *S. lactucae* V105_16, and *Scandinavium* type species

	V105_6^T^	V105_16	*S. goeteborgense* CCUG 66741^T*^	*S. hiltneri* H11S7^T*^	*S. manionii* H17S15^T*^	*S. tedordense* TWS1a^T*^
*Saturated fatty acids*						
C_12:0_	3.0	3.2	2.9	3.2	3.2	3.5
C_14:0_	5.4	5.1	5.2	7.5	7.0	7.4
C_16:0_	32.1	30.5	30.8	31.0	31.1	32.8
*Unsaturated fatty acids*						
C_18:1 ω_7_c_	14.9	16.4	15.6	16.5	16.2	14.7
*Cyclopropane fatty acids*						
C_17:0_ cyclo	10.0	7.5	16.4	14.7	14.7	13.1
*Summed features*						
2: C_14:0_ 3-OH and/or iso-C_16:1_	6.8	7.3	7.7	7.7	7.7	8.1
3: C_16:1_ ω7c and/or C_16:1_ ω6c	20.8	16.4	17.9	16.5	16.3	18.0

*The fatty acid profile of these strains was obtained from a previous study [6]

### Virulence Genes

Our isolates were found to possess 34–43 virulence genes according to the VFDB, including flagella structure, capsule, and a T6SS. The presence of the T6SS genes was further identified with 101–103 different genes against the SecReT6 database. Genes for membrane complex (*tss*J, *tss*M, *tag*L, and *tss*L), spike (PAAR and *tss*I), baseplate (*tss*K, *tss*F, *tss*G, and *tss*E), sheath and tube (*tss*B, *tss*C, and *tss*D), and distal end (*tss*A) proteins were detected in all four isolates.

## Conclusion

The results of phenotypic, biochemical analysis, phylogenetic and genomic analyses indicate that strain V105_6^T^ (= LMG 33389^T^ = DSM 117134^T^) represents a novel species within the genus *Scandinavium*, for which the name *Scandinavium lactucae* sp. nov. is proposed.

### Emendation of Genus *Scandinavium*

The Genus *Scandinavium* is as described by Marathe et al. [2] and the description is based on the data from [2, 4] and this study. Some species in the genus may be ornithine decarboxylase, β-glucuronidase, and α-glucosidase positive. The genus *Scandinavium* has been reported in a wide variety of habitats, and strains have been isolated from human wound infection, rhizosphere soil, bleeding lesions of broadleaf hosts, and lettuce. The major fatty acids are C_16:0_, C_18:1_ ω7*c,* C_17:0_ cyclo, and summed feature 3 (C_16:1_ ω7c and/or C_16:1_ ω6c).

The type species of the genus is *Scandinavium goeteborgense* (CCUG 66741^T^ = CECT 9823^T^ = NCTC 14286^T^).

### Description of *Scandinavium lactucae* sp. nov.

*Scandinavium lactucae* sp. nov. (lac.tu′cae. L. gen. n. *lactucae* of lettuce).

Cells are Gram-negative, catalase positive, rod-shaped and oxidase negative. Growth occurs at 7, 10, and 35 °C, but not at 41 °C. The salt and pH ranges for growth are 1–7% and 5–11, respectively. Colonies on LB agar plate are cream, circular, entire, flat, and 1–2 mm in diameter after 18 h of incubation at 35 °C. Exhibit peritrichous flagella and fimbriae. Positive for ornithine decarboxylase, lysine decarboxylase, β-glucosidase, malonate, β-galactosidase (ONPG), and α-glucosidase. Negative for arginine dihydrolase, urease, L-arabitol, potassium 5-ketogluconate, lipase, D-adonitol, palatinose, indole production, N-acetyl-β-glucosaminidase, saccharose, D-arabitol, α-galactosidase, inositol, and L-aspartic acid arylamidase. Acid is produced from galacturonate, D-mannitol, D-maltose, D-glucose, L-arabinose, D-trehalose, L-rhamnose, and D-cellobiose, and acidification of phenol red. The major fatty acids are C_16:0_, C_18:1_ *ω7c*, C_17:0_ cyclo, and summed feature 3 (C_16:1_ *ω7c* and/or C_16:1_ *ω6c*). The DNA mol% G + C content ranges from 54.32 to 54.56%, with an average genome size is 4.73 Mbp.

The type strain is V105_6^T^ (= LMG 33389^T^ = DSM 117134^T^). The GenBank accession number of the whole genome sequence of the type strain V105_6^T^ is JAWXRD000000000.

The accession numbers at DDBJ/ENA/GenBank for the draft genomes are listed in Table 1.

### Supplementary Information

Below is the link to the electronic supplementary material.

Supplementary file1 (DOCX 123 KB)

Supplementary file2 (DOCX 15 KB)

## Abbreviations

MLSA: Multi-locus sequence analysis
ANI: Average nucleotide identity
dDDH: Digital DNA-DNA hybridization

## Declarations

### Research Involving Human and/or Animal Participants

This article does not contain any studies with animals or human participants.
