# Supplementary material for: Scandinavium lactucae sp. nov. Isolated from Healthy Lettuce in South Korea
Source: Curr Microbiol. 2024 Aug 7;81(9):299. doi: 10.1007/s00284-024-03811-9 (PMC11306268; doi:10.1007/s00284-024-03811-9)
Supplement: Supplementary file 1 — Supplementary file1 (DOCX 123 KB) [file 284_2024_3811_MOESM1_ESM.docx]

Supplementary Figure 1. A phylogenetic tree based on 16S rRNA gene sequences obtained through Sanger sequencing of strains V105_1, V105_6^T^, V105_12, and V105_16, with their related taxa. The tree was constructed by the maximum-likelihood method and Kimura two-parameter model [18]. Numbers at nodes indicate the percentage of 1000 bootstrap replications. The commonly recovered branches in Neighbor-Joining (NJ), Maximum-Likelihood, and Maximum-Parsimony algorithms are depicted by the black circles (⬤). The scale bar indicates 0.01 substitutions per nucleotide position.

Supplementary Figure 2. (a) Fingerprint profile of RAPD-PCR using Pearson correlation (b) Fingerprint profile of rep-PCR using Dice coefficient

Supplementary Figure 3. A heatmap generated using accessory gene data from the pan-genome analysis PANAROO, illustrating the clonal relationships between V105_1 and V105_6^T^, as well as between V105_12 and V105_16.

Supplementary Figure 4. Protein sequence alignment of QnrB variants found in the four isolates with QnrB1 and QnrB96. Black and white color indicate consensus and difference, respectively. Multiple sequence alignment obtained using BLASTp [28].

Supplementary Figure 1

*Scandinavium* sp. V105_1 (PP412027)

*Scandinavium* sp. V105_16 (PP412030)

*Scandinavium* sp. V105_6^T^ (PP412028)

*Scandinavium* sp. V105_12 (PP412029)

*Pluralibacter gergoviae* ATCC 33028^T^ (AB004748)

*Scandinavium manionii* H17S15^T^ (OM987268)

*Scandinavium hiltneri* H11S7^T^ (OM987267)

*Scandinavium goeteborgense* CCUG 66741^T^ (MK558235)

*Scandinavium tedordense* TWS1a^T^ (OM987269)

*Buttiauxella agrestis* ATCC 33320^T^ (AJ293685)

*Leclercia adecarboxylata* NBRC 102595^T^ (AB681872)

*Raoultella planticola* ATCC 33531^T^ (Y17659)

*Cedecea davisae* DSM 4568^T^ (AF493976)

*Lelliottia nimipressuralis* LMG 10245^T^ (Z96077)

*Kluyvera ascorbata* NBRC 102466^T^ (NR114107)

*Gibbsiella quercinecans* FRB 97^T^ (GU562337)

*Citrobacter freundii* LMG 3246^T^ (HG798906)

*Pseudescherichia vulneris* ATCC 33821^T^ (AF530476)

*Yokenella regensburgei* CIP 105435^T^ (JN175339)

*Klebsiella pneumoniae* ATCC 13883^T^ (Y17656)

Siccibacter turicensis LMG 23730T (HQ992947)

*Phytobacter diazotrophicus* DSM 17806^T^ (KY288669)

*Phytobacter massiliensis* JC163^T^ (JN657217)

*Kosakonia cowanii* CIP 107300^T^ (AJ508303)

*Escherichia coli* JCM 1649^T^ (LC069032)

*Shigella dysenteriae* ATCC 13313^T^ (CP026774)

*Enterobacter cloacae* subsp. *cloacae* DSM 30054^T^ (HE978272)

*Salmonella enterica* subsp. *enterica* NBRC 13245^T^ (AB680380)

*Pseudocitrobacter faecalis* DSM 27453^T^ (KF057941)

*Enterobacillus tribolii* IG-V01^T^ (HG972968)

*Trabulsiella guamensis* ATCC 49490^T^ (AY373830)

*Cronobacter sakazakii* ATCC 29544^T^ (EF059843)

*Shimwellia pseudoproteus* 521^T^ (FJ267523)

*Mangroveibacter plantisponsor* MSSRF40^T^ (EF643377)

*Franconibacter helveticus* LMG 23732^T^ (JX986978)

*Izhakiella capsodis* N6PO6^T^ (KF436763)

*Plesiomonas shigelloides* NCIMB9242^T^ (X60418)

*Biostraticola tofi* BF36^T^ (AM774412)

*Rosenbergiella nectarea* 8N4^T^ (HQ284827)

94

51

60

97

0.01

Supplementary Figure 2

a

^T^

b

^T^

Supplementary Figure 3


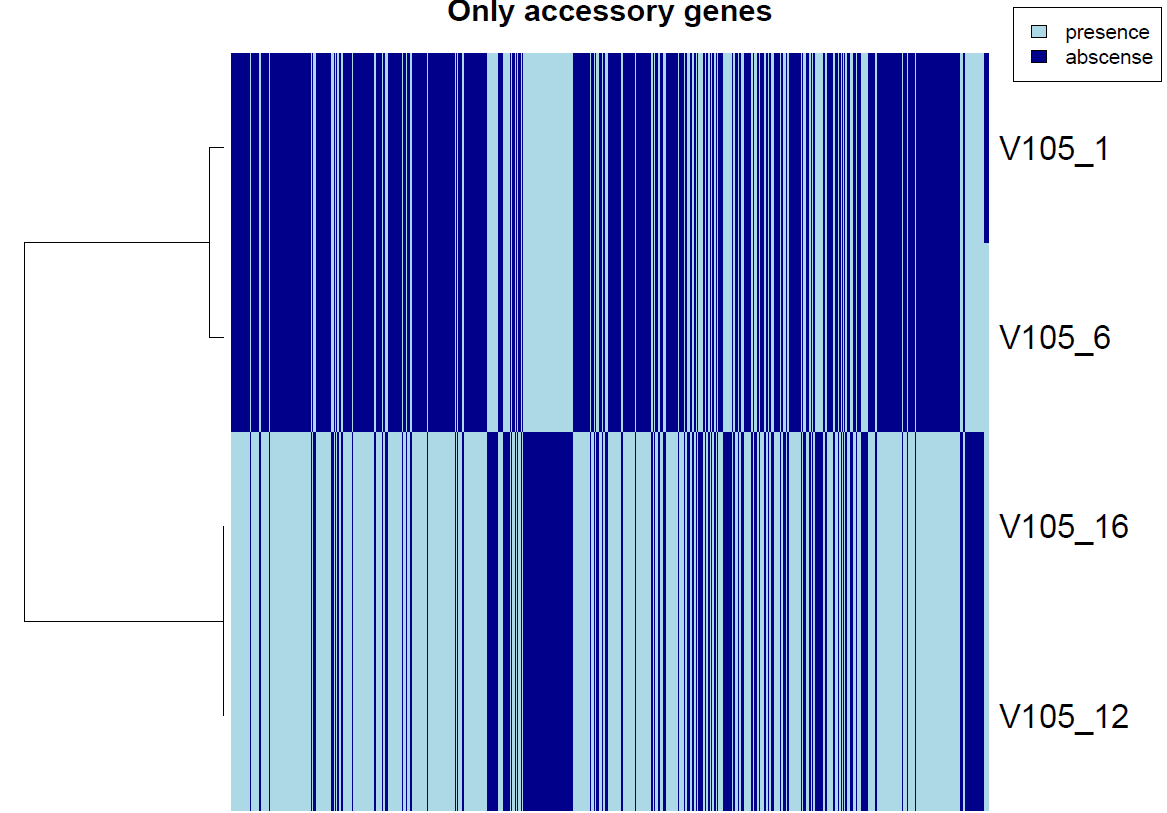


^T^

Supplementary Figure 4

**QnrB1 1** **MALALVGEKIDRNRFTGEKIENSTFFNCDFSGADLSGTEFIGCQFYDRESQKGCNFSRAM**

**QnrB96 1** **MTLALTAEKIERNRFTGLKVENSTFHHCDFSGADLTGTEFIGCQFYDRENQKGCNFSRAI**

**V105_6^T^ 1** **MTLALTAEKIERNRFTGLKVENSTFHHCDFSGADLTGTEFIGCQFYDRESQKGCNFSRAI**

**V105_1 1** **MTLALTAEKIERNRFTGLKVENSTFHHCDFSGADLTGTEFIGCQFYDRESQKGCNFSRAI**

**V105_12 1** **MTLALTAEKIERNRFTGLKVENSTFHHCDFSGADLTGTEFIGCQFYDRESQKGCNFSRAI**

**V105_16 1** **MTLALTAEKIERNRFTGLKVENSTFHHCDFSGADLTGTEFIGCQFYDRESQKGCNFSRAI**

**QnrB1 61** **LKDAIFKSCDLSMADFRNSSALGIEIRHCRAQGADFRGASFMNMITTRTWFCSAYITNTN**

**QnrB96 61** **LKDAIFKNCDLSMADFRNASALGIEIRGCRAQGADFRGTSFMNMITTRTWFCSAYITNTN**

**V105_6^T^ 61** **LKDAIFKDCDLSMADFRNASALGIEIRGCLAQGSDFRGTSFMNMITTRTWFCSAYITNTN**

**V105_1 61** **LKDAIFKDCDLSMADFRNASALGIEIRGCLAQGSDFRGTSFMNMITTRTWFCSAYITNTN**

**V105_12 61** **LKDAIFKDCDLSMADFRNASALGIEIRGCLAQGSDFRGTSFMNMITTRTWFCSAYITNTN**

**V105_16 61** **LKDAIFKDCDLSMADFRNASALGIEIRGCLAQGSDFRGTSFMNMITTRTWFCSAYITNTN**

**QnrB1 121** **LSYANFSKVVLEKCELWENRWIGAQVLGATFSGSDLSGGEFSTFDWRAANFTHCDLTNSE**

**QnrB96 121** **LSYANFSKAVLEKCELWENRWMGTQVLGATFSGSDLSGGEFSSFDWRAANVTHCDLTNSE**

**V105_6^T^ 121** **LSYANFSKAVLEKCELWENRWIGTQILGATFSGSDLSGGEFSSFDWRAANVTHCDLTNSE**

**V105_1 121** **LSYANFSKAVLEKCELWENRWIGTQILGATFSGSDLSGGEFSSFDWRAANVTHCDLTNSE**

**V105_12 121** **LSYANFSKAVLEKCELWENRWIGTQILGATFSGSDLSGGEFSSFDWRAANVTHCDLTNSE**

**V105_16 121** **LSYANFSKAVLEKCELWENRWIGTQILGATFSGSDLSGGEFSSFDWRAANVTHCDLTNSE**

**QnrB1 181** **LGDLDIRGVDLQGVKLDNYQASLLMERLGIAVIG**

**QnrB96 181** **LGDLDVRGVDLQGVKLDSYQVSLIMERLGVAIIG**

**V105_6^T^ 181** **LGDLDVRSVDLQGVKLDSYQVSLIMDRLGIAIIG**

**V105_1 181** **LGDLDVRSVDLQGVKLDSYQVSLIMDRLGIAIIG**

**V105_12 181** **LGDLDVRSVDLQGVKLDSYQVSLIMDRLGIAIIG**

**V105_16 181** **LGDLDVRSVDLQGVKLDSYQVSLIMDRLGIAIIG**
