# Supplementary material for: Scandinavium lactucae sp. nov. Isolated from Healthy Lettuce in South Korea
Source: Curr Microbiol. 2024 Aug 7;81(9):299. doi: 10.1007/s00284-024-03811-9 (PMC11306268; doi:10.1007/s00284-024-03811-9)
Supplement: Supplementary file 2 — Supplementary file2 (DOCX 15 KB) [file 284_2024_3811_MOESM2_ESM.docx]

| Antibiotics  Strain Number | **CN 10** | **TOB 10** | **CIP 5** | **S 10** | **C 30** | **AMP 10** | **FOX 30** | **MEM 10** | **CTX 30** | **TET 30** |
| --- | --- | --- | --- | --- | --- | --- | --- | --- | --- | --- |
| **1. V105_6^T^** | - | - | i | - | - | + | i | - | - | - |
| **2. V105_1** | - | - | - | i | - | + | + | - | i | - |
| **3. V105_12** | - | - | i | - | - | + | + | - | - | - |
| **4. V105_16** | - | - | - | - | - | + | + | - | - | - |

Supplementary Table 1. Antibiotic susceptibility profiles of strains V105_1, V105_6^T^, V105_12, and V105_16 as determined by disc diffusion assays and classified based on CLSI guidelines. Resistant (+); Intermediate (i); Susceptible (-). Ampicillin (AMP), cefoxitin (FOX), cefotaxime (CTX), chloramphenicol (C), ciprofloxacin (CIP), gentamicin (CN), erythromycin (E), meropenem (MEM), sulfonamides (S), tetracycline (TE), and tobramycin (TOB).
